# Comparative Analysis of Chemical Descriptors by Machine Learning Reveals Atomistic Insights into Solute–Lipid Interactions

**DOI:** 10.1021/acs.molpharmaceut.4c00080

**Published:** 2024-05-23

**Authors:** Justus Johann Lange, Andrea Anelli, Jochem Alsenz, Martin Kuentz, Patrick J. O’Dwyer, Wiebke Saal, Nicole Wyttenbach, Brendan T. Griffin

**Affiliations:** † School of Pharmacy, University College Cork, College Road, Cork T12 R229, Cork County, Ireland; ‡ Roche Pharma Research and Early Development, Therapeutic Modalities, Roche Innovation Center Basel, F. Hoffmann-La Roche Limited, Grenzacherstrasse 124, Basel 4070, Switzerland; § Insitute of Pharma Technology, University of Applied Sciences and Arts Northwestern Switzerland, Hofackerstrasse 30, Muttenz CH-4231, Basel City, Switzerland

**Keywords:** smooth overlap of atomic positions (SOAP), machine learning, solubility prediction, lipids, lipid based formulations, quantitative-structure−property-relationships (QSPR)

## Abstract

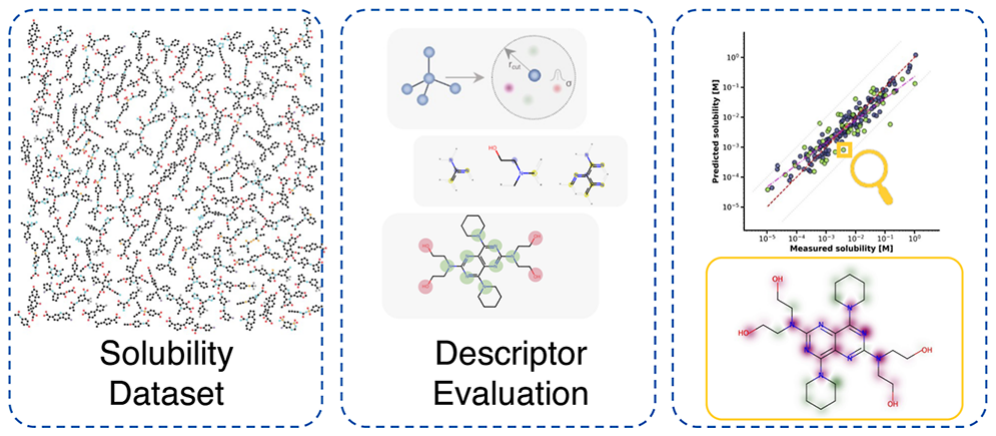

This study explores the research area of drug solubility in lipid excipients, an area persistently complex despite recent advancements in understanding and predicting solubility based on molecular structure. To this end, this research investigated novel descriptor sets, employing machine learning techniques to understand the determinants governing interactions between solutes and medium-chain triglycerides (MCTs). Quantitative structure-property relationships (QSPR) were constructed on an extended solubility data set comprising 182 experimental values of structurally diverse drug molecules, including both development and marketed drugs to extract meaningful property relationships. Four classes of molecular descriptors, ranging from traditional representations to complex geometrical descriptions, were assessed and compared in terms of their predictive accuracy and interpretability. These include two-dimensional (2D) and three-dimensional (3D) descriptors, Abraham solvation parameters, extended connectivity fingerprints (ECFPs), and the smooth overlap of atomic position (SOAP) descriptor. Through testing three distinct regularized regression algorithms alongside various preprocessing schemes, the SOAP descriptor enabled the construction of a superior performing model in terms of interpretability and accuracy. Its atom-centered characteristics allowed contributions to be estimated at the atomic level, thereby enabling the ranking of prevalent molecular motifs and their influence on drug solubility in MCTs. The performance on a separate test set demonstrated high predictive accuracy (RMSE = 0.50) for 2D and 3D, SOAP, and Abraham Solvation descriptors. The model trained on ECFP4 descriptors resulted in inferior predictive accuracy. Lastly, uncertainty estimations for each model were introduced to assess their applicability domains and provide information on where the models may extrapolate in chemical space and, thus, where more data may be necessary to refine a data-driven approach to predict solubility in MCTs. Overall, the presented approaches further enable computationally informed formulation development by introducing a novel in silico approach for rational drug development and prediction of dose loading in lipids.

## Introduction

The process of identifying the most suitable formulation for a drug candidate is increasing in complexity and requires careful decision-making, primarily due to the high prevalence of poorly water-soluble drug candidates.^1,2^ These drugs often require more sophisticated formulation strategies, termed bioenabling approaches, to improve their absorption and consequently their bioavailability.^3^ Preformulation profiling plays a pivotal role in this context. This involves extensive solubility screenings in a diverse range of excipients, which provide the basis for formulation development.^4^ This step is crucial in understanding the challenges associated with a given drug candidate and tailoring effective formulations. The commercial reality of reducing time-to-market and the need to move away from property-agnostic formulation development underscore the importance of computationally informed approaches. Leveraging *in silico* methods can mitigate trial and error in formulation development and support informed decision-making in drug product development.^5−8^ Lipid-based formulations are a bioenabling approach with demonstrated clinical success.^9^ This formulation strategy typically involves constructing complex phase diagrams based on solubility screenings in specific solvents. Adopting in silico approaches in this process can be beneficial as it can enable decision-making based on molecular properties. Through the extraction of chemical insights by computational approaches, the formulation process can be streamlined, accelerated, and better understood.

Over the past decade, data-driven approaches have made significant strides in predicting solubility in formulation vehicles utilizing quantitative structure property relationships (QSPR).^10^ Conceptually, the accuracy of any machine learning model depends on the quality of the data set, the algorithm used, as well as the way in which molecular properties are being encoded. Pioneering research in understanding and predicting solubility in triglycerides has been conducted utilizing various modeling techniques and features such as two-dimensional (2D) and three-dimensional (3D) descriptors, as well as solvation parameters.^11−14^ Different classes of descriptors portray molecular properties in different formats, and there is arguably not one set of “best” descriptors that captures all drivers of solubilization in lipids.^15^

Previous works on predicting drug solubility in medium-chain triglycerides (MCTs) focused on 2D and 3D descriptors to construct linear regression models via partial least-squares regression modeling.^12^ It was found that descriptors related to the solid state, in the form of calculated ideal solubility, as well as the polarity, size, and shape of the molecule, are of relevance to predicting solubility in MCTs. While chemical representations of molecules in the form of topological polar surface area (TPSA) and charge distribution certainly facilitate a better understanding of the factors driving solvation, such global molecular determinants have limited application to providing an atomistic understanding of the factors at play. For example, the number of nitrogen atoms was previously identified to be of relevance; however, such count-based descriptors convey shortcomings in terms of accounting for mesomeric and inductive effects exerted by chemical proximity.^11,12^

Studies focusing on more mechanistic aspects of drug solubility and partitioning in lipidic excipients used Abraham solvation parameters for the construction of linear free energy relationships (LFER).^14,16^ These descriptors comprise a collection of five numerical values that encode a molecular structure by considering its molar volume, solute H-bond acidity and basicity, as well as excess molar refraction and polarity/polarizability.^17^ The application of these descriptors as Abraham-type LFER equations successfully demonstrated their effectiveness in predicting solubility enhancement by fasted-state-simulated intestinal fluid.^18^ The ease of employing Abraham descriptors and their succinct way to represent molecular properties has facilitated widespread use to model several partition equilibria and biological properties.^15,17,19^

Most of the descriptors mentioned above assign chemical information (i.e., polarity or H-bonding strength) to structural characteristics. However, molecular fingerprints, such as extended connectivity fingerprints (ECFPs), describe atomic environments based on the presence or absence of substructures within a predefined bond length. This encoding method captures the connectivity aspects of molecules.^20^ While the predominant application of ECFPs focuses on similarity searching, recently, ECFPs have been utilized to predict drug solubility in organic solvents and water.^21,22^

A more complex class of geometrical fingerprints focused on atomic densities has recently seen a surge of applications in the field of materials modeling.^23^ These descriptors create parametrizable descriptions of the local spatial regions composing an atomistic system, providing accurate structural information on their targets common molecular fingerprints. The smooth overlap of atomic position descriptors (SOAP) in particular has performed convincingly in many prediction tasks oriented at characterizing the stability of organic compounds, both in condensed and gas phase applications,^24,25^ with remarkable generalization performances.^26^ The constructed regression model assigned stability attributes to local spatial regions within a molecule through physicochemically motivated machine learning.^27^

One notable advantage of utilizing such encodings lies in the direct interpretability of the atom-centered regression weights in the context of their impact on the target property under investigation. Unlike approaches that interpret global molecular determinants, such as calculated logP, this method facilitates an understanding of how spatial regions of a molecule contribute to the modeled property. This aspect gains particular significance within the domain of advancing explainable artificial intelligence (AI).^6,28^ Models that offer explanations regarding the mapping of input features to the target property are more widely accepted and trusted by users and may be utilized to better understand the property being investigated.

There are many different ways to represent molecular structures. These encompass a broad spectrum of attributes, spanning from physicochemical characteristics to complex geometric descriptions. By leveraging the data resources available from preclinical profiling repositories, machine learning holds the potential to improve predictive capabilities, provide novel insights into the governing principles of solubility in MCTs, and thereby further supplement the current understanding of the underlying factors at play.^5^

The objective of this study was to compare and evaluate various descriptor sets with a specific emphasis on SOAP descriptors. The predictive accuracy, interpretability, and uncertainty of each set were assessed using an extended data set of solubility values in MCTs. This approach offers the opportunity to move away from local models and instead uncover global trends in solubility by capturing a larger chemical space.^29^ This approach allows for a more comprehensive understanding and broader practical insights.

## Materials and Methods

### Materials

Miglyol 812 N (MCT; IOI Oleo GmbH, Hamburg, Germany) was purchased from Warner Graham. The excipient complies with the quality specifications of the European Pharmacopoeia. The solvents used for the ultra-performance liquid chromatography (UPLC) quantification were of UPLC grade.

#### Data Set Characteristics

For this study, a data set of solubility values for 182 crystalline drugs in MCTs was curated. Out of the 182 molecules, 51 violate the rule of five defined by Lipinski et al.,^30^ and 72 molecules correspond to development compounds by F. Hoffmann-La Roche Ltd. The solubility is provided as the decadic logarithm of the molar solubility (log*S*). Descriptive statistics of common physicochemical properties and their underlying distribution are presented in Table 1. The data set does not contain any multicomponent crystals, e.g., salts, hydrates, or solvates. The experimental data for the compounds used in this study (MP, solubility in MCTs) can be found in the Supporting Information.

**Table 1:** Descriptive Statistics of Common Physicochemical Properties (*n* = 182)

statistics	mean	std	min	**25%**^a^	median	**75%**^a^	max
TPSA [Å^2^]^b^	71.81	31.98	6.48	46.53	71.85	90.84	182.83
*c*log*P*^c^	3.78	1.73	–1.04	2.73	3.67	4.71	8.90
*M*_w_ [g mol^–1^]^b^	396.08	111.88	151.17	314.77	389.37	458.31	764.95
MP [°C]	175.14	54.13	55.47	138.12	172.35	216.90	302.00
logS [M]	–2.30	1.02	–4.98	–3.02	–2.33	–1.56	0.04

a 25th and 75th percentile.

b Calculated by *RDKit*.

c Calculated by *RDKit* according to Wildman and Crippen.^31^

### Methods

#### Solubility Measurements and Data Curation

Drug solubility in MCTs was determined by (a) mixing the samples for 24 h at room temperature by using a miniaturized 96-well assay for solubility and residual solid screening (SORESOS),^32^ (b) employing a miniaturized version of the shake-flask method in 2 mL glass vials,^33^ or (c) collecting data from the literature.^12,34^ Each of the employed assays involved residual solid-state screenings by powder X-ray diffraction to identify potential solid-state changes during solubility screenings.

#### Thermophysical Analysis

The melting point of the drugs was determined as the onset of the melting endotherm by differential scanning calorimetry (DSC), recorded with a DSC I instrument from Mettler-Toledo AG (Greifensee, Switzerland). Thermogravimetric analysis (TGA) was employed to confirm the absence of solvates or hydrates and to ensure that no degradation occurs during the DSC heat ramps. Samples were analyzed with a TGA/DSC 1 STARe system from Mettler-Toledo AG (Greifensee, Switzerland). Both DSC and TGA measurements were performed as described previously.^35^

### Descriptor Calculation and Model Construction

#### RDKit—Mol File Generation and ECFP Calculation

*RDKit* is an open-source cheminformatics software which provides a range of functions for working with chemical structures and data.^36^*RDKit* (Version 2022.9.5) was employed for the calculation of ECFPs via the Morgan algorithm, creation of mol files, molecular embedding, and chemical structure representation. Mol files were obtained based on simplified molecular-input-line-entry system sequences (SMILES).^37^ ECFPs are a class of connectivity fingerprints that encode structural fragments of a molecule, considering attached bonds and atoms within a defined circular bond distance.^20^ Each molecule was encoded, considering a distance of 2 or 3 bonds as 2048 bits. ECFP fingerprints are frequently utilized for similarity analysis of compound libraries; however, to the best of the authors’ knowledge, this set of features has never been introduced to model drug solubility in lipids.

#### Mordred—2D and 3D Descriptors

2D and 3D descriptors were calculated with *Mordred*, an open-source descriptor software (Version 1.2.0),^38^ based on previously generated .mol files via *RDKit*, 1826 2D and 3D descriptors were calculated. The success of these calculations relies on the specific SMILES sequence provided as input. In certain cases, the calculation process did not calculate all descriptors successfully. For that reason, non-numeric features were excluded from the dataframe, which resulted in 1218 descriptors that were further preprocessed. Collinear descriptors can be assumed to contain redundant information. To address modeling issues arising from collinearity, a threshold of ≥95% was applied, leading to the exclusion of features surpassing this threshold. It is important to emphasize that the identification of cross-correlated features was performed by using statistics from the training set and then extended to the test set. This approach was adopted to avoid any potential bias introduced by train-test leakage.^6^

#### DScribe—Smooth Overlap of Atomic Positions Descriptor

*DScribe* is an open-source Python package, initially developed for material sciences purposes, which allows for the transformation of atomic structures to numerical fingerprints.^39,40^ Throughout this study, it was used to calculate the SOAP descriptor (*DScribe* version 2.0.0). The SOAP descriptor encodes the atomic environment of each atom in a molecule by estimating the probability density of other atoms residing at specific distances relative to a focal atom, yielding a geometrical fingerprint for each atom within a molecule. The granularity of this description depends highly on its parametrization and should be optimized by a target-adapted regression approach to adequately reflect the properties influencing the dependent variable. Spatial geometries for each atom within a molecule are iteratively encoded by optimizing the parameters *r*_cut_, *l*_max_, *n*_max_, σ, and the averaging mode. The *r*_cut_ parameter represents a cutoff radius in Å, which takes the contribution of each atomic species to the environment for each focal atom into account. Any atom residing outside the defined radius is neglected during the calculation. The *n*_max_ and *l*_max_ parameters predominantly define the dimensionality of the descriptor, specifying the local expansion, and correspond to the number of radial basis functions and the maximum degree of spherical harmonics used to describe the atomic environments, respectively. This can be considered as the resolution of the environment defined within the cutoff *r*_cut_. Finally, the σ value represents the width of a Gaussian that represents the atomic density fields for each atom in the system. Within this study, values for *r*_cut_ from 5 to 20 (increment = 1), for *n*_max_ and *l*_max_ from 2 to 10 (increment = 2), and σ from 0.1 to 1.5 (increment = 0.1) comparable to Barnard et al.^41^ were evaluated. For each of these combinations, a separate model was trained. The generated features represent a tensor that must be averaged to be suitable for the algorithms employed herein. Every molecule within the data set was embedded to assign each atom to 3D coordinates. The .xyz files were read with the *Atomic Simulation Environment* (*ASE*) (Version 3.22.1) and passed to *DScribe* for further processing.^42^ The optimal SOAP parameters were determined as part of a 10-fold cross-validation scheme on the training set. The model with the lowest root mean squared error (RMSE) obtained on average within 10-fold was chosen for further evaluation.

#### AbSolv—Abraham Solvation Parameters

For the calculation of Abraham solvation parameters (AbSolv Descriptors), the software Percepta, implemented in ACD Laboratories (Advanced Chemistry Development, Inc. Toronto, Canada) was utilized [ACD/Laboratories Release 2021.2.2 (Build 3535. Seventeen Dec 2021)]. The calculation was based on SMILES sequences.

#### Model Building Procedure

Each model was constructed using *scikit-learn*, an open-source machine learning library.^43^ To develop and evaluate the models, a consistent train-test split was used. The dataframe was sorted by log solubility (mol L^–1^), prior to assigning every fourth compound to the test set, resulting in a training set consisting of 75% of the data. This guaranteed that the chemical features influencing solubility were distributed evenly in both sets. Within this study, the feature matrices included the melting point as a variable to address the impact of solid-state characteristics on solubility.

Each model was trained using K-fold cross-validation (*K* = 10) on the training set to optimize the hyperparameters of each model. *Scikit-learn* pipelines and grid searches were utilized to avoid train-test leakage. To ensure that the features and models compared are assessed equally, shuffling within the cross-validation scheme was conducted with the same random seed. Preprocessing steps were fitted to the training set and used to transform the data in the test set. These involved the evaluation of different scaling methods, such as the *MinMaxScaler*, *StandardScaler*, and *RobustScaler*(43) and skewness transformation via the *yeo-johnson* method, which effectively maps features to normal distributions.^44^ The selection of the final estimator for further evaluation was guided by choosing the model that achieved the lowest RMSE within the cross-validation scheme. This estimator was finally evaluated on the yet unseen test set. This approach was adopted to mitigate the potential influence of a fortuitous train-test split on the model selection process. By prioritizing the estimator with the lowest cross-validated RMSE, which accounts for performance across multiple train-validation splits, a more reliable and robust evaluation of the model’s generalization ability and predictive performance was obtained.

To address modeling challenges posed by the high dimensionality of the data set, regularized linear methods, i.e., least absolute shrinkage and selection operator (lasso), ridge, and elastic net regression were evaluated. Lasso is a linear regression, technique that applies an L1 penalty term during optimization. This term facilitates sparsity in the model by shrinking the coefficients, resulting in few nonzero coefficients. It is an effective method to prevent overfitting and efficiently select the most predictive features, which promotes a more interpretable and compact model.^45,46^ Similarly, ridge regression promotes shrinking the coefficients by applying an L2 penalty term, lowering the coefficients but never forcing them to be zero.^47^ While lasso is particularly suited for feature selection purposes, the ridge L2 penalty offers an effective strategy to deal with high collinearity. Both methods are combined in the elastic net model, which employs both the L1 and L2 penalties, offering more flexibility in controlling the sparsity and overall complexity of the resulting model.^46,48,49^ The tunable hyperparameters of the models are the α value, which controls the regularization strength, as well as the L1 to L2 ratio for the elastic net model. This study aims to obtain useful models regarding predictive accuracy while targeting meaningful descriptors for drug solubility in lipids, rather than aiming to exhaustively test different algorithms. For this reason, relatively simple but interpretable regression frameworks have been investigated that allow good comparison between models while being computationally inexpensive. Although ridge is well suited for dealing with multicollinearity, highly correlated features were excluded from the feature frame based on training set statistics when utilizing 2D and 3D descriptors. To evaluate the model performance, RMSE, mean absolute error (MAE), and R^2^ were considered. The same metrics were calculated and reported for a leave-one-out cross validation (LOOCV) on the training set. The external test set was used for final estimator evaluation only and remained unseen during training.

### Uncertainty and Applicability Domain Estimation

To estimate uncertainties associated with the input variables, several models were aggregated based on different subsamples of the training data.^23^ For a total of 1000 iterations, each model with its predetermined hyperparameters was fitted to a ratio of 90% of the training data. This partition was sampled at random from the training set without replacement. The different fits on the subset were utilized to predict the test instances to derive a point estimate with an associated standard deviation. This corresponds to the uncertainty of the model for a given instance and reflects an estimate of the feature space where the model is likely to inter- and extrapolate. The generation of subsamples was conducted by using the numpy random.choice function.^50^ A calibration of uncertainties as well as rescaling of the predicted distribution was conducted according to Imbalzano et al.^51^ Ultimately, this approach enabled the quantification of uncertainties associated with each model’s predictions, providing insights into the reliability and applicability domain (AD) of each model and descriptor set. As highlighted by Musil et al.,^52^ such an approach facilitates deriving conclusions on a feature or molecular space, in which the training set lacks sufficient input space to derive highly reliable predictions.

## Results and Discussion

In this study, the performance of four different descriptor sets to predict solubility in MCTs was evaluated by the application of three regularized regression approaches and various preprocessing schemes on a data set consisting of 182 experimental solubility values. To accommodate the impact of solid-state contributions to solubility, MP was included as a feature in all descriptor sets under evaluation. The results for the obtained models are summarized in Table 2. Based on the test set statistics, the features show comparable predictive performance. However, when both cross-validation results and interpretability are considered, the SOAP descriptor emerges as the most suitable choice for predicting solubility in MCTs.

**Table 2:** Performance for the Best Model per Descriptor Set^a^

feature performance				
feature	2D and 3D	SOAP	Abraham	ECFP4
Train Performance				
*R*^2^	0.82	0.92	0.70	0.88
RMSE	0.42	0.28	0.55	0.35
MAE	0.33	0.21	0.42	0.27
Test Performance				
*R*^2^	0.77	0.78	0.77	0.68
RMSE	0.50	0.49	0.50	0.59
MAE	0.41	0.42	0.38	0.45
LOOCV on Training Set				
*Q*^2^	0.68	0.72	0.66	0.65
RMSE	0.57	0.53	0.58	0.60
MAE	0.43	0.42	0.44	0.45

a Model type and hyperparameters can be inferred from Table S2.

### Modeling with 2D and 3D Descriptors

**Figure 1:**
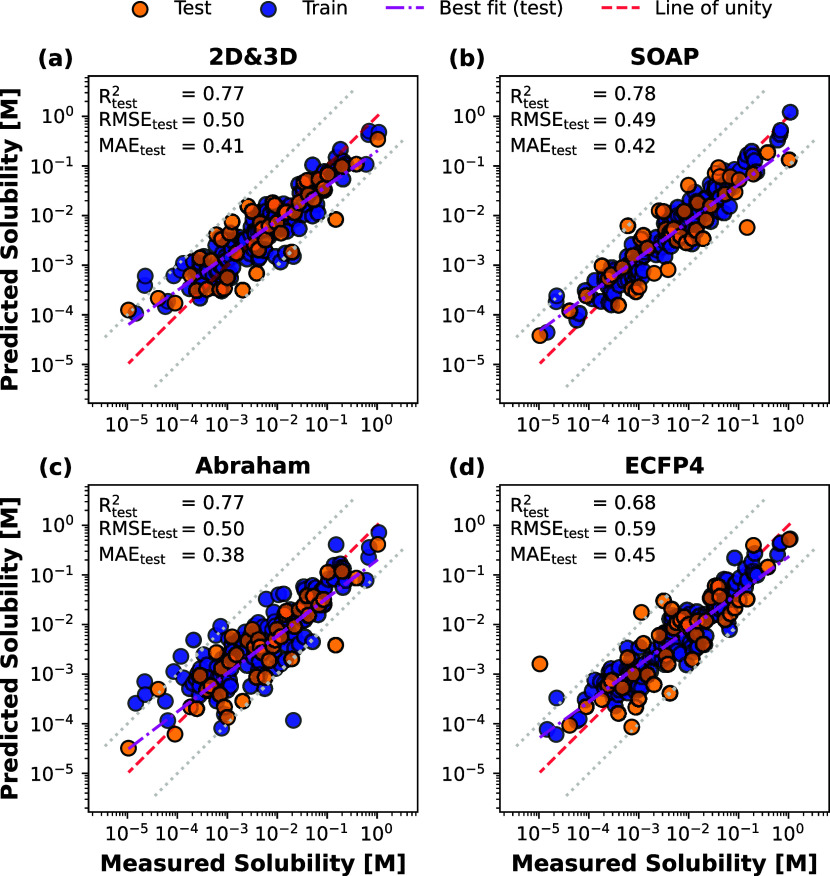
Parity plots illustrate the comparison between the predicted and measured solubility for 182 drugs in MCTs by the application of different descriptors and machine learning pipelines. The gray dotted lines represent a deviation of ±0.5 log_10_ units from the identity line. The line of best fit on the test set is plotted to assess the models’ average deviation from unity.

2D and 3D descriptors are among the most abundantly used features for QSPR modeling and have demonstrated successful application in modeling various properties.^18,21,22,53,54^ Application of the *Mordred* open-source descriptor library followed by the previously outlined preprocessing methodology resulted in a performance of *R*^2^ = 0.77 and an RMSE = 0.50 on the test set (Table 2). A parity plot, displayed in Figure 1a illustrates the performance of the model on the training and test splits. Clearly, the model accurately predicts drug solubility in MCTs. Yet, it is notable that the model exhibited a lower degree of accuracy in predicting the solubility of compounds with a low solubility in MCTs. This is evident by an overprediction of solubility for those compounds. Conducting leave-one-out cross-validation on the training set resulted in an RMSE of 0.57, which is overall in agreement with the test performance, both indicating good generalizability on unseen data. The previously described preprocessing scheme resulted in a model input consisting of 822 features after removing cross-correlated features based on training set statistics. As a part of the constructed machine learning pipeline, the impact of skewed variables and the effects of scaling and centering were assessed. The pipeline that resulted in the most robust cross-validated accuracy on the training set was chosen. Among the models tested on 2D and 3D descriptors, the elastic net model with prior feature transformation via the *MinMaxScaler* yielded the highest performance. The *MinMaxScaler* transforms the scale of each feature to values between 0 and 1. Skewness transformation by employing the *yeo-johnson* transformer did not result in superior robustness, as indicated by a higher RMSE during cross-validation. Hyperparameter tuning resulted in an α-value of 11.51 × 10^–3^ and an L1 ratio of 0.75. For a full overview of the model performance, the reader is directed to Table 2.

**Figure 2:**
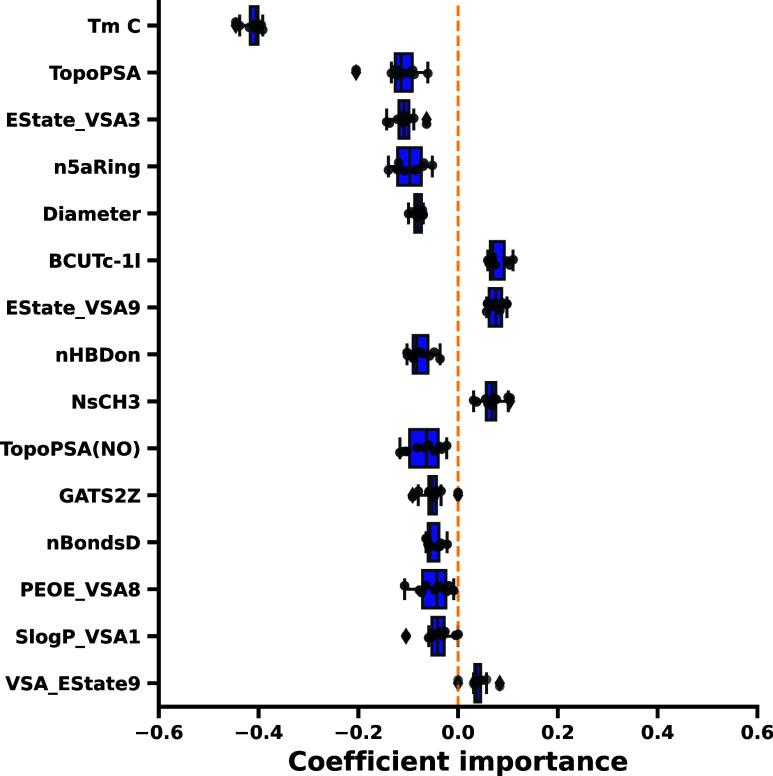
Coefficient values across a 10-fold cross-validation on the training set of the transformed features. The 15 most influential features for solubility in MCTs are displayed. Most features exhibit a negative influence on solubility. Solid-state properties, reflected by the melting point, appear to be most influential for the trained model. An explanation of the used abbreviations can be inferred from the Supporting Information (Table S2).

An assessment of feature importance was conducted by considering the coefficient values of the model across a 10-fold cross-validation scheme on the training set. Figure 2 illustrates which solute properties influence solubility in MCTs by considering the models’ regression weights. It is well known that the solid-state properties of the drugs are a major driving force for solubility in lipid excipients such as MCTs, which is further reaffirmed by the negative coefficient value.^8^ The solute’s melting point, serving as a surrogate for the crystal lattice energy required for the molecule’s dissociation from the crystal, constitutes the most prominent predictor with a negative influence in the elastic net model. The molecule’s polarity, expressed as TPSA, had a negative influence but was of lower priority.^55^ This is further reflected by the effect of various electrotopological state (E-State) indices that represent numerical values comprising topology and local electron accessibility of the molecular structures.^56^ Essentially, the van der Waals surface area (VSA) number to the E-State index can be considered as the surface contribution of a certain part of the molecule to the global E-State index of the molecule. The number of aromatic five-membered rings (n5aRing) represents the feature with the fourth highest predictivity when considering the coefficients of the model. Five-membered rings are often encountered as nitrogen-containing heterocycles that introduce further polarity into the molecule, which may consequently lead to a negative impact on solubility in lipids. Further indicators of the relevance of polarity and electrotopology included the “Burden Chemical Abstract Service University of Texas” (BCUT) descriptors weighted by Gasteiger-Marsilli partial charges or partial equalization of orbital electronegativity (PEOE).^57−60^

The importance of the polarity of the solute to predict lipid solubility aligns with observations using a linear regression approach for a set of 34 drugs based on 2D and 3D descriptors.^11,12^ Among the most predictive models, the TPSA (tot) and the melting point were identified as important features, alongside the number of double bonds and the number of nitrogen atoms. Additionally, the JGI6 descriptor positively influenced lipid solubility, which is a topological descriptor reflecting the global charge transfer within a molecule.^12,61,62^ Although other descriptors representing electrotopology were identified by the discussed machine learning model, it can be concluded that similar trends were observed.

The machine learning model developed in this study exhibits a marginally lower level of accuracy than the model developed by Alskär et al.^12^ for predicting solubility in MCTs when considering the RMSE on the test set (RMSE = 0.50 (*n* = 46) vs RMSE = 0.37 (*n* = 6)). This may be linked to the larger chemical diversity present in the current data set (*n* = 182 vs *n* = 35) and also the larger solubility interval. Another explanation may be that only the melting point was utilized as a proxy for solid-state contributions and not the ideal solubility, which accounts for the entropy of fusion calculated based on the enthalpy of fusion and the melting point.^12^ A previous study by the same authors resulted in a model with a weaker RMSE of 0.75 (*n* = 8) when only MP was included as an additional feature.^11^

### Modeling with Smooth Overlap of Atomic Position Descriptors

The SOAP descriptor encodes the local atomic environment of each atomic species encountered within a molecule.^63^ Depending on its parametrization, the descriptor can offer a highly granular view on atomic topology and connectivity and has been recently applied to predict diverse properties such as aqueous solubility and the stability of organic molecular crystals.^27,41^ The SOAP descriptor was initially designed for material sciences purposes, and its utility to model solubility in pharmaceutically relevant solvents has yet to be explored.

The construction of a SOAP tensor starts with a 3D embedding of a molecular structure, which is followed by an extraction of atomic species present within a molecule. For each atom within the molecule a probability density of locating other atoms in a user defined proximity of each focal atom is being calculated (*r*_cut_ in Å). The parameters *l*_max_ and *n*_max_ define the resolution of the encoded 3D space that falls within the vector length of *r*_cut_. Finally, each atom is represented by the standard deviation of the Gaussian used to expand the atomic density. A more detailed explanation of the descriptor is provided by Himanen et al.^39^ and Bartók et al.^63^ The calculation of the SOAP descriptor yielded tensors of different dimensionality, dependent on the choice of parameters. The maximum accuracy in this study was achieved by summing the values of the computed tensor over each atom. The parameters for the SOAP descriptor that rendered the highest predictive performance are *r*_cut_ = 5, *n*_max_ = 8, *l*_max_ = 2, and σ = 0.3, which resulted in 6240 values describing local atomic environments of each atom.

Among the different regularized models evaluated, the lasso algorithm yielded the highest accuracy within the 10-fold cross-validation scheme (α-value = 2.95 × 10^–2^). The resulting feature matrix, including MP, was scaled by the *StandardScaler*, which involved removing the mean and scaling to unit variance. A parity plot is shown in Figure 1b that presents the relationship between measured and predicted drug solubility in MCTs.

Important in any data driven approach is not only the performance on new unseen data but also the causal inferences that can be drawn between features and target properties.^28^ As the SOAP descriptors encode local environments for each atom in a molecule, it allows iteratively decomposing a global property such as solubility to atomic contributions. This can provide insights into solute–lipid interactions on an atomistic level. For this purpose, a separate model was built and fitted on the average of the descriptors associated with its individual atomic species. The local atomic environment for each atomic species was used as an input to predict the contribution of each atom toward the total solubility. These contribution values were indexed by subtracting the total solubility value, enabling the identification of atomic environments that positively or negatively impacted the solubility.

**Figure 3:**
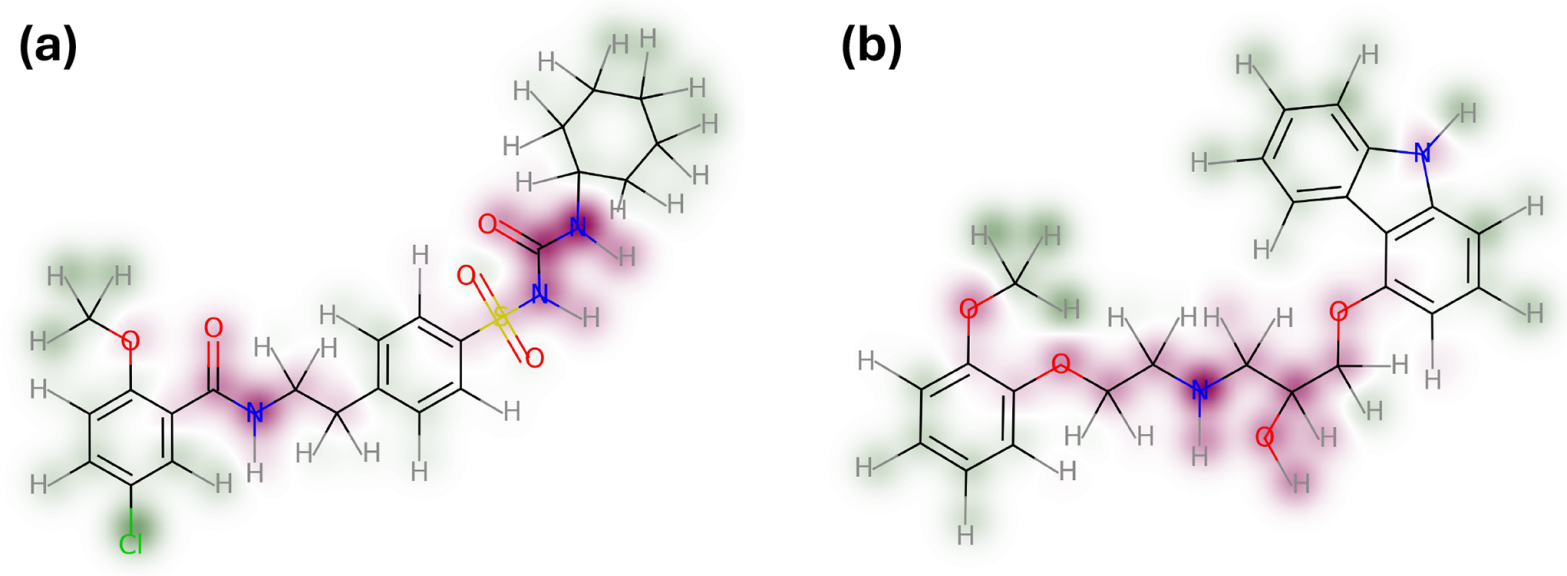
Contribution maps for the model drugs a) glibenclamide and b) carvedilol. Atoms highlighted in green demonstrate a positive impact on solubility in lipids, while red atoms contribute negatively. The contributions are indexed by the predicted solubility for the molecules, which constitutes the average of all atomic predictions.

Figure 3a,b presents highlighted contributions for two different molecules by utilizing the RDKits GetSimilarityMapFromWeights function.^36^ The example for the model drug glibenclamide (Figure 3a) clearly shows that atomic environments inducing polarity negatively influenced the solubility in lipids. The sulfonylurea substructure of glibenclamide considerably influences solubility, likely owing to the presence of free electron pairs within the functional group and its NH-acidic character. The machine learning model successfully recognized patterns in the atomic environments, as reflected by the negative contributions of adjacent substructures. For example, the neighboring benzene moiety appears to be influenced by the sulfonylurea structure, resulting in a reduction of its positive effect. This demonstrates the model’s ability to capture complex relationships and interactions of atomic environments within a molecule.

As a consequence, atoms in the ortho position exhibit no discernible effect, which sets them apart from other benzene rings. The amide functional group of glibenclamide exerted a similar negative contribution. Interestingly, the amide adjacent to the ethyl group constitutes a negative impact, most likely attributed to the polarity of the amide. It should be noted that the SOAP descriptor was calculated on a 3D embedding of the molecule. Considering the 2D structure of the molecule, it can be observed that due to the presence of a rotatable bond, the amide structure and ethyl moiety may exhibit spacial proximity that might have been taken into account during the prediction. This is confirmed by the conformation of the embedded molecule (not shown). The cyclohexane moiety, as well as a high share of the benzene moieties, had a positive influence on solubility, which is most likely attributed to the increased lipophilicity these functional groups trigger. It should be noted that the machine learning model used the MP as a feature that results in challenges to deconvolute properties from solvation or solid-state limited solubility. It can be suspected that the MP accounted for a high proportion of the crystal lattice energy term that must be surmounted for the molecule to dissociate from the crystal. For that reason, the model predominantly identified solute–solvent interactions, as opposed to dissociation of solute–solute interactions.

As an example of another drug, carvedilol was chosen to represent atomic contributions, which are visualized in Figure 3b. The strong negative contribution resulting from the diethylamine moiety exhibits a considerably higher contribution compared to the H-bond donor of the carbazole system as the free electron pair is likely to delocalize into the ring system. Attributing these contributions to structural motifs further underscores the potential of local decompositions of a global property such as solubility.

**Figure 4:**
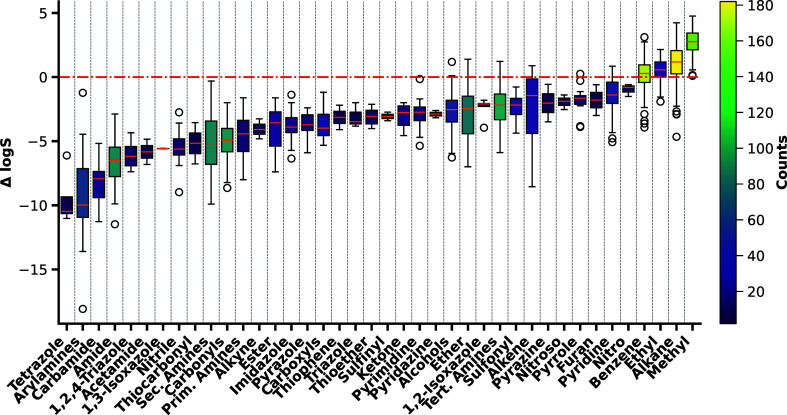
Boxplot depicting the average normalized contributions to solubility per functional group. The box spans from the first to the third quartile, with a median line, and whiskers that extend 1.5 times the interquartile range. The observed high variability may be due to electron delocalization and contrasting contributions from overlapping atomic environments. Only functional groups appearing in two or more molecules were included in the analysis.

To make the results more widely applicable, an attempt was made to delineate the atomic contributions by functional group, although the examples of glibenclamide and carvedilol show that the atomic geometries influencing solubility overlap. A list of SMILES arbitrary target specification (SMARTS) patterns was utilized to extract the atomic contributions driving solubility via substructure matching. The contributions per functional group were averaged. A descriptive analysis of the obtained results is depicted in Figure 4.

**Figure 5:**
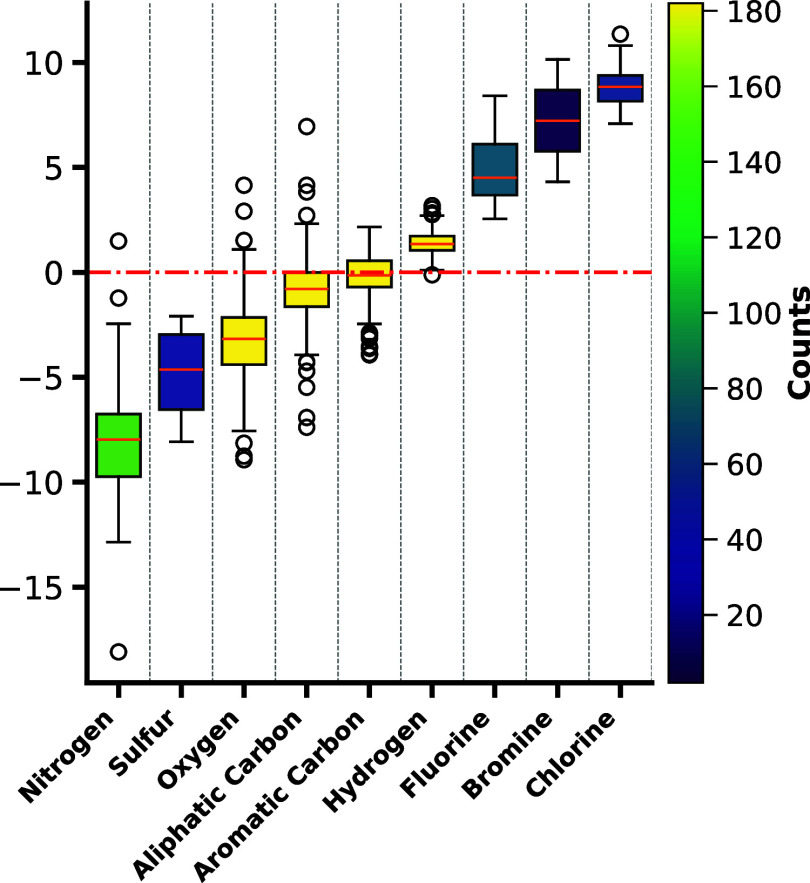
Boxplot of average contributions to lipid solubility per atom, normalized and highlighted by the number of molecules in the data set that contain each atom.

The contributions of substructures showed noticeable variability, mainly due to the diverse proximal environment of these functional groups, which was highlighted based on the examples carvedilol and glibenclamide. Nevertheless, a clear differentiation between substructures that enhance solubility and those that hinder solubility was evident. The positive influence of benzene rings and hydrocarbons is reaffirmed by the model’s indication that benzene, ethyl, and methyl are located within the solubility-enhancing region of the boxplot. Considering the contributions per atomic species, it was noticeable that halogens appeared to have a favorable impact on solubility in MCTs, particularly chlorine and fluorine (Figure 5).

Both of these atoms were sufficiently represented in the molecular structures, as opposed to bromine. Substituting hydrogens with fluorine atoms is a concept frequently utilized in drug discovery to enhance permeability through lipid membranes by increasing lipophilicity while minimizing the increase in atomic radius.^64^ Hence, the positive impact may be attributed to the increase in lipophilicity facilitated by the introduction of these atoms. An evident negative impact was observed for tetrazole rings, arylamines, carbamides, and amide moieties. While this finding highlights the importance of nitrogen atoms that were previously identified to negatively impact solubility,^11,12^ the atomistic perspective provided by the application of the SOAP descriptor demonstrated the shortcomings of this approach as it appears that nitrogen atoms can exert very diverse properties, depending on their atomic environment, which is reflected by the high variation.

### Modeling with Abraham Solvation Parameters

Abraham solvation parameters have shown great promise for mechanistic investigations of drug partitioning and solubility in MCTs.^13,14,16^ However, they have never been utilized for a data-driven assessment of drug solubility in MCTs. The underlying theory of the Abraham solvation parameters is related to the framework of the cavity model, which describes solute–solvent interactions.^65^ This study does not aim to build classical linear free energy relationships but rather utilizes the 5 Abraham parameters and includes MP to reflect solid-state properties as a part of the previously described machine learning pipeline. The best model obtained with this descriptor set was a ridge regression model with an α-value of ≈0.494 and preprocessing steps involving the previously elaborated *MinMaxScaler* and skewness transformation via the *yeo-johnson* method, which effectively transforms skewed variables to normal distributions.

The performance metrics of this approach are displayed in Table 2 and Figure 1c. Contrary to expectations, the utilization of Abraham solvation descriptors resulted in a lower performance on the training set, with an RMSE of 0.55, compared to an RMSE of 0.50 on the test set. This finding may be attributed to a fortuitous train-test split, resulting in features and weights that favor generalizing to the test set. It should also be noted that the computation of Abraham solvation parameters relies on the availability of established molecular fragments with well-defined associated values. Yet, in the case of development compounds featuring novel molecular motifs, certain fragments may be inadequately represented during the descriptor calculation. Consequently, there is a possibility that the used values for these novel fragments are imprecise, thereby failing to accurately capture the properties of these groups. This could falsify the overall value for a certain feature. In fact, the ACD/Laboratories AbSolv algorithm is based on group contributions developed by Platts et al.^66^ with additional optimizations, and it was previously noted that particular substructures led to larger prediction errors when modeling solvent/water partition coefficients.^67^ Especially, halogenated and bridged compounds led to larger prediction errors, which highlights the shortcomings of descriptors calculated based on group contribution approaches, where insufficient calibrations might not be available.^15^

Apart from the McGowan volume, all feature importances suggested a negative impact on solubility in lipids based on the coefficients of the model (data not shown). Most relevant were the H-bonding basicity and the solid-state properties reflected by the MP. Hydrogen bond acidity influenced solubility in lipids to a lower magnitude. This may be attributed to a trade-off between the polarity that arises from the atoms constituting the donor of the molecule and a previously reported beneficial impact on partitioning into lipidic excipients by solute–solvent complexation between the esters of glyceride moieties and drugs.^13^

**Figure 6:**
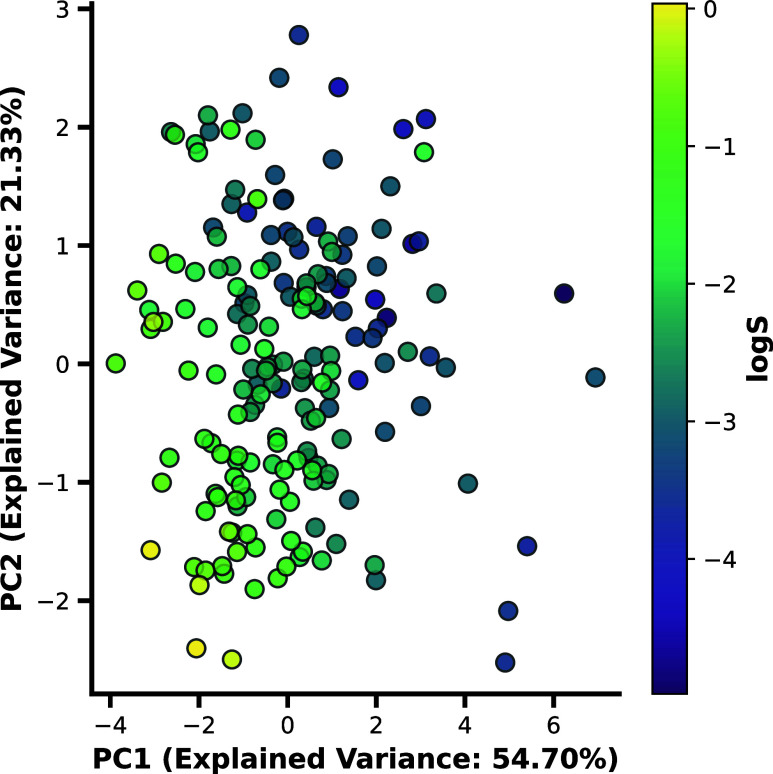
First two principal components of the PCA explain 76.03% of the variance in the data. This decomposition allows for the identification of a clear trend for solubility in lipids based on Abraham solvation parameters and MP.

Overall, the Abraham solvation parameters rendered similar predictivity compared to the 2D and 3D descriptors considering the RMSE during CV. In Figure 6, a PCA of the Abraham solvation descriptors, including MP, color-mapped by solubility of the molecule, is provided. The figure reveals a good differentiation between highly and poorly soluble drugs.

### Modeling with Extended Connectivity Fingerprints

ECFPs are widely used for similarity searching of drug-like molecular libraries and have also been explored for predicting drug properties, including solubility in aqueous and organic solvents.^20−22^ These descriptors can be parametrized by different cutoff values that specify the bond length considered to assign bits to neighboring structural attributes. The resulting 2048 bit values specify whether a particular substructure is present or not. In most cases, bit radii of two or three are considered that correspond to ECFP4 and ECFP6, respectively.

The best-performing model utilizing ECFPs was an elastic net model without feature scaling or transformations (Figure 1d). The model employed an α-value of 2.95 × 10^–2^ and an L1 ratio of 0.25. Despite applying a penalty function, the relatively low L1 ratio may have contributed to slight overfitting and inadequate regularization, as evidenced by the high performance on the training set and comparatively lower performance on the testing set (Table 2). The low L1 ratio might also be explained by the limited chemical information that these features convey, which implies a rather limited need for regularization. The achieved *R*^2^ value of 0.68 on the test set was lower than the *R*^2^ obtained using other descriptors in this study. Moreover, the cross-validated RMSE showed higher values and greater fluctuation, suggesting that the features used in this model may lack robust predictability. The utilization of ECFP6 did not yield a higher predictive performance compared to ECFP4 (data not shown). In conclusion, while ECFPs have proven useful for similarity searching of drug-like molecules, their efficacy for predicting drug properties, such as solubility in MCTs, appears limited when compared to alternative descriptors. This is underscored by a recent study attempting aqueous solubility prediction with ECFPs, which concluded that many common machine learning algorithms do not construct metavariables, in the form of hidden layers that could capture more complex interfeature relationships and relate them to solubility.^22^ It can be assumed that the predictive capabilities for ECFPs will only be notable when considering larger data sets. Statistical inferences drawn between the presence or absence of a substructure and its corresponding effect are more difficult to assign when training on binary features compared to continuous descriptors, as reflected by the performance metrics obtained with 2D and 3D descriptors. It should be noted that the decomposition of a global molecular property such as solubility of solutes in lipids by considering molecular fragments lacks an adequate description of effects arising from inductive and mesomeric effects as well as different conformations, which is inferior to the SOAP descriptor. Additionally, ECFPs contain only one identifier per fragment, which could lead to an insufficient representation of repeated fragments.^20^

### Uncertainty and Applicability Domain Estimation

**Figure 7:**
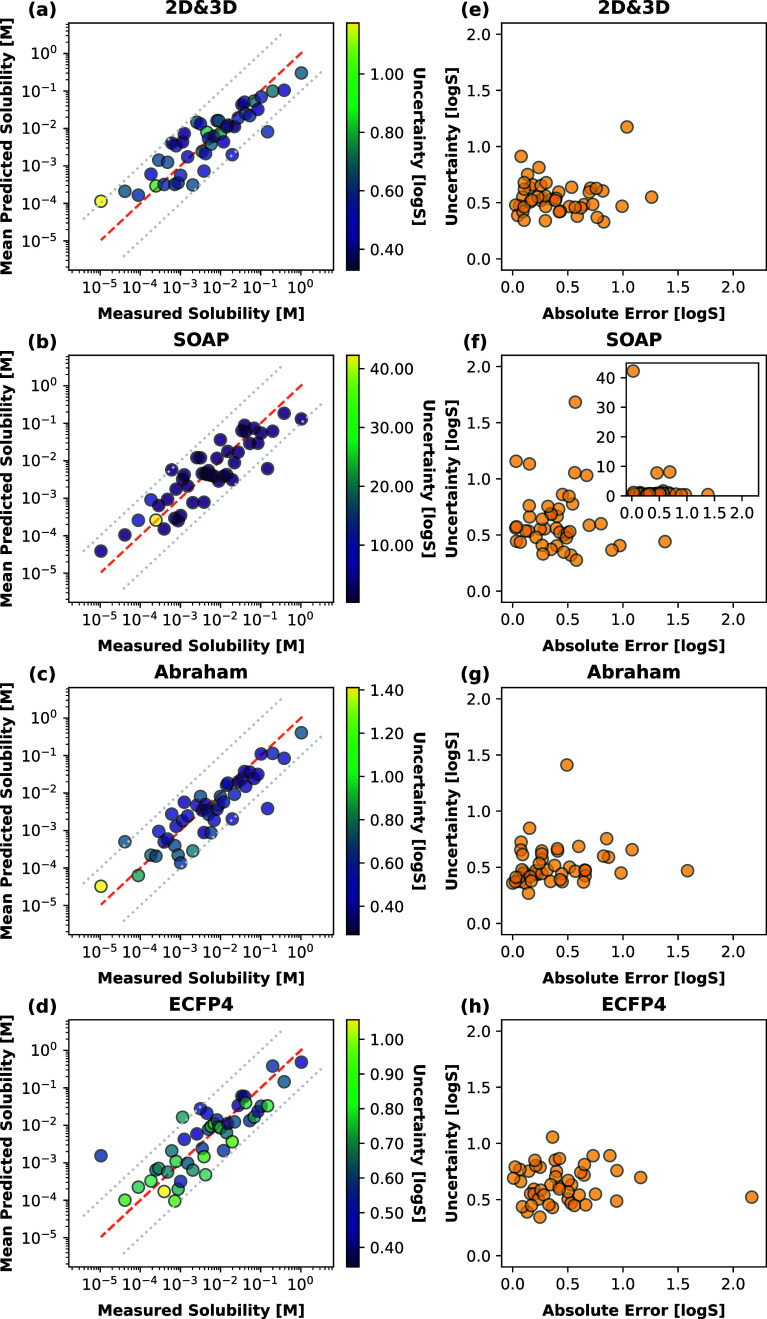
(a–d) Parity plots illustrating the performance of bagged models on the test set, each trained on subsamples of the training data. The uncertainties are expressed as standard deviation around the mean value and visualized using color mapping. It is important to note the differing scales of the colorbars, emphasizing the degree of uncertainty among the models. (e–h) Uncertainty plotted over the absolute error.

To gauge the uncertainties associated with each model and establish an estimate for their applicability domains, we aggregated a collection of 1000 models was aggregated. These models were fitted using subsampling on the training data, with hyperparameters specified, as outlined in Table S1. Subsequently, this ensemble of models was used to make predictions for the test set. Figure 7a–d illustrates the performance of the bagged models by plotting the predicted mean for each instance derived from the 1000 different fits and mapping the standard deviation for each prediction to each instance after calibration and rescaling.^51^

The results demonstrate that the aggregated models were successful in generalizing to the test set. High standard deviations for a given molecule reflect that the corresponding feature space may be underrepresented in the training set, as might be the case for molecules with a relatively distinctive chemical structure. This serves as a surrogate for the underlying applicability domain and potential extrapolations of the model.

The results obtained based on the bagged models on 2D and 3D descriptors are depicted in Figure 7a, and an overview on the relation between uncertainty and absolute error is illustrated in Figure 7e. A certain degree of uncertainty is inherent to a model as it can always be considered as a local construct. The five molecules with the highest uncertainties for each model/descriptor are depicted in Figure S1. Notably, high prediction uncertainties were associated with molecules such as digitoxin, hydrochlorothiazide, and colchicine, which exhibit relatively distinctive chemical structures. This underscores the connection between training set diversity and the model’s ability to make reliable predictions, highlighting areas where the model’s applicability domain may be limited.

For the model built on the SOAP descriptor, overall higher uncertainties were obtained, as depicted in Figure 7b,f, with three outliers present. It appears that the model constructed on SOAP descriptors is more sensitive to training data selection and that within certain feature spaces; more training data would be required to provide more reliable predictions. However, as indicated by the low absolute error associated with these structures, the bag of models was still capable of predicting the solubility value for the provided structures successfully. Notably, among the structures with the highest uncertainty were again hydrochlorothiazide and digitoxin, which is comparable to the model built on 2D and 3D descriptors, in addition to RO5114497-000 and RO5014449-000, two research compounds. The high uncertainties obtained from the model based on SOAP descriptors may be attributed to the fact that the SOAP descriptor provides the most granular description of an atomic system among the descriptors investigated. If the algorithm was not provided with adequate information on these particular systems during subsampling, it may inherently fail to capture these instances while generalizing on unseen data.

As illustrated in Figure S1, the model built on Abraham Solvation descriptors was in agreement with these results, as the highest uncertainties were again associated with digitoxin as well as the same two research compounds. The analysis of the model using ECFP4 descriptors indicated that the uncertainties associated with predictions for different compounds were relatively consistent for a specific lead series within the data set. This may suggest a model bias toward these instances, potentially due to their prevalent representation in the training set.

A point of concern arises with compounds exhibiting low uncertainty yet displaying high absolute errors in their predictions. This discrepancy could indicate that, while the model suggests it gives a reliable prediction for certain chemical spaces, which are well represented in the training set, it may not accurately capture the true solubility values, particularly when these values deviate significantly in the test set. Such observations might suggest a need to reassess the model’s regularization and, by that, investigate potential overfitting. This was also indicated by the considerable difference between the train and test performance in Table 2 for the ECFP4 features. The estimation of uncertainties highlights the importance of not only evaluating model performance based on average accuracy metrics but also examining individual prediction uncertainties and errors to uncover subtle biases and areas for model improvement. Building separate models and different clusters of the data may also display merits going forward, once sufficient amounts of data are available.^26^ Generally, such uncertainty estimations can be utilized to further refine a model by providing it with more data in the chemical space it may be lacking, thereby providing a potential avenue for active learning.^52^

### Comparison of Machine Learning Approaches to Thermodynamic Modeling and Quantum Chemistry

Finally, the predictive performance of the results above should be compared to predictive models outside of data-driven methodologies. Recent works on the application of thermodynamic modeling via the perturbed-chain statistical associating fluid theory (PC-SAFT) demonstrated successful application to identify more complex formulation compositions that were mostly in agreement with experimental categorization according to the lipid-based classification system.^68−70^ A recent study employed the conductor-like screening model for real solvents (COSMO-RS) theory, utilizing the COSMOquick software.^34^ An MAE of 0.576 on a logarithmic scale by using a simplified lipid approach was achieved. The results in Table 2 emphasize the promising prospects of data-driven methodologies for predicting pharmaceutically relevant properties by machine learning, as the models reported herein surpass the predictive performance obtained from more complex polarization charge densities derived from statistical thermodynamics and quantum chemistry.

## Conclusions

This study provides a novel atomistic view of structural characteristics involved in solute–triglyceride interactions by the utilization of machine learning. The decomposition of the global solute property solubility was achieved by assigning solvation contributions to atomic environments by an atom-centered regression approach using the SOAP descriptor. This sheds light on the interplay between molecular structure and solubility behavior. Benchmarking the SOAP descriptor against more conventional descriptors further highlights their advantage in facilitating an understanding of solubility. The estimation of uncertainties by the utilization of a committee of models highlights in which chemical space the models may give less reliable predictions and whether they may inter- or extrapolate, which may increase the trust of users in the model. The findings of this study pave the way for more informed decision-making in the development of solubility-tailored formulations. Further applications of the SOAP descriptor could be considered to investigate additional pharmaceutically relevant properties, as it offers novel perspectives beyond 2D and 3D descriptors. It is recommended to extend its use to calculate spatial atomic geometries within periodic systems such as molecular crystals as a model input that could show promise in reducing the strong reliance on solid-state characteristics such as MP.

## Supporting Information Available

Information table for the reported models, including hyperparameters as well as applied preprocessing steps; explanation of most influential 2D and 3D descriptors; and depiction of the five molecules exhibiting the highest uncertainties associated with their predicted solubility per descriptor/model (PDF)
  Solubility data set including SMILES sequences and MP for 174 molecules (XLSX)

## References

[ref1] Ditzinger F.; Price D. J.; Ilie A.-R.; Köhl N. J.; Jankovic S.; Tsakiridou G.; Aleandri S.; Kalantzi L.; Holm R.; Nair A.; Saal C.; Griffin B.; Kuentz M. Lipophilicity and hydrophobicity considerations in bio-enabling oral formulations approaches – a PEARRL review. J. Pharm. Pharmacol. 2019, 71, 464–482. 10.1111/jphp.12984. 30070363

[ref2] Bergström C. A.; Charman W. N.; Porter C. J. Computational prediction of formulation strategies for beyond-rule-of-5 compounds. Adv. Drug Delivery Rev. 2016, 101, 6–21. 10.1016/j.addr.2016.02.005. 26928657

[ref3] O’Driscoll C.; Griffin B. Biopharmaceutical challenges associated with drugs with low aqueous solubility—The potential impact of lipid-based formulations. Adv. Drug Delivery Rev. 2008, 60, 617–624. 10.1016/j.addr.2007.10.012. 18155800

[ref4] Alsenz J.; Kansy M. High throughput solubility measurement in drug discovery and development. Adv. Drug Delivery Rev. 2007, 59, 546–567. 10.1016/j.addr.2007.05.007. 17604872

[ref5] Reppas C.; Kuentz M.; Bauer-Brandl A.; Carlert S.; Dallmann A.; Dietrich S.; Dressman J.; Ejskjaer L.; Frechen S.; Guidetti M.; et al. Leveraging the use of in vitro and computational methods to support the development of enabling oral drug products: An InPharma commentary. Eur. J. Pharm. Sci. 2023, 188, 106505 10.1016/j.ejps.2023.106505. 37343604

[ref6] Murray J. D.; Lange J. J.; Bennett-Lenane H.; Holm R.; Kuentz M.; O’Dwyer P. J.; Griffin B. T. Advancing Algorithmic Drug Product Development: Recommendations for Machine Learning Approaches in Drug Formulation. Eur. J. Pharm. Sci. 2023, 191, 106562 10.1016/j.ejps.2023.106562. 37562550

[ref7] Kuentz M.; Holm R.; Kronseder C.; Saal C.; Griffin B. T. Rational Selection of Bio-Enabling Oral Drug Formulations – A PEARRL Commentary. J. Pharm. Sci. 2021, 110, 1921–1930. 10.1016/j.xphs.2021.02.004. 33609523

[ref8] Kuentz M.; Holm R.; Elder D. P. Methodology of oral formulation selection in the pharmaceutical industry. Eur. J. Pharm. Sci. 2016, 87, 136–163. 10.1016/j.ejps.2015.12.008. 26687443

[ref9] Bennett-Lenane H.; O’Shea J. P.; O’Driscoll C. M.; Griffin B. T. A Retrospective Biopharmaceutical Analysis of 800 Approved Oral Drug Products: Are Drug Properties of Solid Dispersions and Lipid-Based Formulations Distinctive?. J. Pharm. Sci. 2020, 109, 3248–3261. 10.1016/j.xphs.2020.08.008. 32822721

[ref10] Bennett-Lenane H.; O’Shea J. P.; Murray J. D.; Ilie A.-R.; Holm R.; Kuentz M.; Griffin B. T. Artificial Neural Networks to Predict the Apparent Degree of Supersaturation in Supersaturated Lipid-Based Formulations: A Pilot Study. Pharmaceutics 2021, 13, 1398 10.3390/pharmaceutics13091398. 34575483 PMC8466847

[ref11] Persson L. C.; Porter C. J. H.; Charman W. N.; Bergström C. A. S. Computational Prediction of Drug Solubility in Lipid Based Formulation Excipients. Pharm. Res. 2013, 30, 3225–3237. 10.1007/s11095-013-1083-7. 23771564 PMC3841656

[ref12] Alskär L. C.; Porter C. J. H.; Bergström C. A. S. Tools for Early Prediction of Drug Loading in Lipid-Based Formulations. Mol. Pharm. 2016, 13, 251–261. 10.1021/acs.molpharmaceut.5b00704. 26568134 PMC4928820

[ref13] Cao Y.; Marra M.; Anderson B. D. Predictive Relationships for the Effects of Triglyceride Ester Concentration and Water Uptake on Solubility and Partitioning of Small Molecules into Lipid Vehicles. J. Pharm. Sci. 2004, 93, 2768–2779. 10.1002/jps.20126. 15389678

[ref14] Rane S. S.; Anderson B. D. What determines drug solubility in lipid vehicles: Is it predictable?. Adv. Drug Delivery Rev. 2008, 60, 638–656. 10.1016/j.addr.2007.10.015. 18089295

[ref15] Livingstone D. J. The Characterization of Chemical Structures Using Molecular Properties. A Survey. J. Chem. Inf. Comput. Sci. 2000, 40, 195–209. 10.1021/ci990162i. 10761119

[ref16] Rane S. S.; Cao Y.; Anderson B. D. Quantitative Solubility Relationships and the Effect of Water Uptake in Triglyceride/Monoglyceride Microemulsions. Pharm. Res. 2008, 25, 1158–1174. 10.1007/s11095-007-9500-4. 18095145

[ref17] Abraham M. H.; Le J. The correlation and prediction of the solubility of compounds in water using an amended solvation energy relationship. J. Pharm. Sci. 1999, 88, 868–880. 10.1021/js9901007. 10479348

[ref18] Niederquell A.; Kuentz M. Biorelevant Drug Solubility Enhancement Modeled by a Linear Solvation Energy Relationship. J. Pharm. Sci. 2018, 107, 503–506. 10.1016/j.xphs.2017.08.017. 28864357

[ref19] Abraham M. H. The factors that influence permeation across the blood–brain barrier. Eur. J. Med. Chem. 2004, 39, 235–240. 10.1016/j.ejmech.2003.12.004. 15051171

[ref20] Rogers D.; Hahn M. Extended-Connectivity Fingerprints. J. Chem. Inf. Model. 2010, 50, 742–754. 10.1021/ci100050t. 20426451

[ref21] Ye Z.; Ouyang D. Prediction of small-molecule compound solubility in organic solvents by machine learning algorithms. J. Cheminf. 2021, 13, 98 10.1186/s13321-021-00575-3. PMC8665485 34895323

[ref22] Lovrić M.; Pavlović K.; Žuvela P.; Spataru A.; Lučić B.; Kern R.; Wong M. W. Machine learning in prediction of intrinsic aqueous solubility of drug-like compounds: Generalization, complexity, or predictive ability?. J. Chemom. 2021, 35, e3349 10.1002/cem.3349.

[ref23] Musil F.; Grisafi A.; Bartók A. P.; Ortner C.; Csányi G.; Ceriotti M. Physics-Inspired Structural Representations for Molecules and Materials. Chem. Rev. 2021, 121, 9759–9815. 10.1021/acs.chemrev.1c00021. 34310133

[ref24] Bartók A. P.; De S.; Poelking C.; Bernstein N.; Kermode J. R.; Csányi G.; Ceriotti M. Machine learning unifies the modeling of materials and molecules. Sci. Adv. 2017, 3, e1701816 10.1126/sciadv.1701816. 29242828 PMC5729016

[ref25] Wengert S.; Csányi G.; Reuter K.; Margraf J. T. A Hybrid Machine Learning Approach for Structure Stability Prediction in Molecular Co-crystal Screenings. J. Chem. Theory Comput. 2022, 18, 4586–4593. 10.1021/acs.jctc.2c00343. 35709378 PMC9281391

[ref26] Zeni C.; Anelli A.; Glielmo A.; Rossi K. Exploring the robust extrapolation of high-dimensional machine learning potentials. Phys. Rev. B 2022, 105, 165141 10.1103/physrevb.105.165141.

[ref27] Cersonsky R. K.; Pakhnova M.; Engel E. A.; Ceriotti M. A data-driven interpretation of the stability of organic molecular crystals. Chem. Sci. 2023, 14, 1272–1285. 10.1039/D2SC06198H. 36756329 PMC9891366

[ref28] Wellawatte G. P.; Gandhi H. A.; Seshadri A.; White A. D. A Perspective on Explanations of Molecular Prediction Models. J. Chem. Theory Comput. 2023, 19, 2149–2160. 10.1021/acs.jctc.2c01235. 36972469 PMC10134429

[ref29] Kuentz M.; Bergström C. A. Synergistic Computational Modeling Approaches as Team Players in the Game of Solubility Predictions. J. Pharm. Sci. 2021, 110, 22–34. 10.1016/j.xphs.2020.10.068. 33217423

[ref30] Lipinski C. A.; Lombardo F.; Dominy B. W.; Feeney P. J. Experimental and computational approaches to estimate solubility and permeability in drug discovery and development settings. Adv. Drug Delivery Rev. 1997, 23, 3–25. 10.1016/S0169-409X(96)00423-1. 11259830

[ref31] Wildman S. A.; Crippen G. M. Prediction of Physicochemical Parameters by Atomic Contributions. J. Chem. Inf. Comput. Sci. 1999, 39, 868–873. 10.1021/ci990307l.

[ref32] Wyttenbach N.; Alsenz J.; Grassmann O. Miniaturized Assay for Solubility and Residual Solid Screening (SORESOS) in Early Drug Development. Pharm. Res. 2007, 24, 888–898. 10.1007/s11095-006-9205-0. 17372689

[ref33] Wyttenbach N.; Niederquell A.; Ectors P.; Kuentz M. Study and Computational Modeling of Fatty Acid Effects on Drug Solubility in Lipid-Based Systems. J. Pharm. Sci. 2022, 111, 1728–1738. 10.1016/j.xphs.2021.11.023. 34863971

[ref34] Alsenz J.; Kuentz M. From Quantum Chemistry to Prediction of Drug Solubility in Glycerides. Mol. Pharm. 2019, 16, 4661–4669. 10.1021/acs.molpharmaceut.9b00801. 31518142

[ref35] Wyttenbach N.; Kirchmeyer W.; Alsenz J.; Kuentz M. Theoretical Considerations of the Prigogine–Defay Ratio with Regard to the Glass-Forming Ability of Drugs from Undercooled Melts. Mol. Pharm. 2016, 13, 241–250. 10.1021/acs.molpharmaceut.5b00688. 26587865

[ref36] Landrum G. RDKit: Open-Source Cheminformatics Software; GitHub, 2016. https://github.com/rdkit/rdkit/releases/tag/Release_2016_09_4.

[ref37] Weininger D. SMILES, a chemical language and information system. 1. Introduction to methodology and encoding rules. J. Chem. Inf. Comput. Sci. 1988, 28, 31–36. 10.1021/ci00057a005.

[ref38] Moriwaki H.; Tian Y.-S.; Kawashita N.; Takagi T. Mordred: a molecular descriptor calculator. J. Cheminf. 2018, 10, 4 10.1186/s13321-018-0258-y. PMC5801138 29411163

[ref39] Himanen L.; Jäger M. O.; Morooka E. V.; Federici Canova F.; Ranawat Y. S.; Gao D. Z.; Rinke P.; Foster A. S. DScribe: Library of descriptors for machine learning in materials science. Comput. Phys. Commun. 2020, 247, 106949 10.1016/j.cpc.2019.106949.

[ref40] Laakso J.; Himanen L.; Homm H.; Morooka E. V.; Jäger M. O. J.; Todorović M.; Rinke P. Updates to the DScribe library: New descriptors and derivatives. J. Chem. Phys. 2023, 158, 234802 10.1063/5.0151031. 37338028

[ref41] Barnard T.; Tseng S.; Darby J. P.; Bartók A. P.; Broo A.; Sosso G. C. Leveraging genetic algorithms to maximise the predictive capabilities of the SOAP descriptor. Mol. Syst. Des. Eng. 2023, 8, 300–315. 10.1039/D2ME00149G.

[ref42] Hjorth Larsen A.; Jørgen Mortensen J.; Blomqvist J.; Castelli I. E.; Christensen R.; Dułak M.; Friis J.; Groves M. N.; Hammer B.; Hargus C.; et al. The atomic simulation environment—a Python library for working with atoms. J. Phys.: Condens. Matter 2017, 29, 273002 10.1088/1361-648x/aa680e. 28323250

[ref43] Pedregosa F.; et al. Scikit-learn: Machine Learning in Python. J. Mach. Learn. Res. 2011, 12, 2825–2830.

[ref44] Yeo I.-K. A new family of power transformations to improve normality or symmetry. Biometrika 2000, 87, 954–959. 10.1093/biomet/87.4.954.

[ref45] Tibshirani R. Regression Shrinkage and Selection Via the Lasso. J. Roy. Stat. Soc. B 1996, 58, 267–288. 10.1111/j.2517-6161.1996.tb02080.x.

[ref46] Kuhn M.; Johnson K. Applied Predictive Modeling; Springer: New York, 2013.

[ref47] Hoerl A. E.; Kennard R. W. Ridge Regression: Biased Estimation for Nonorthogonal Problems. Technometrics 1970, 12, 55–67. 10.1080/00401706.1970.10488634.

[ref48] Zou H.; Hastie T. Regularization and Variable Selection Via the Elastic Net. J. Roy. Stat. Soc. B Stat. Methodol. 2005, 67, 301–320. 10.1111/j.1467-9868.2005.00503.x.

[ref49] Zou H.; Hastie T. Addendum: Regularization and Variable Selection Via the Elastic Net. J. Roy. Stat. Soc. B Stat. Methodol. 2005, 67, 768 10.1111/j.1467-9868.2005.00527.x.

[ref50] Harris C. R.; Millman K. J.; van der Walt S. J.; Gommers R.; Virtanen P.; Cournapeau D.; Wieser E.; Taylor J.; Berg S.; Smith N. J.; et al. Array programming with NumPy. Nature 2020, 585, 357–362. 10.1038/s41586-020-2649-2. 32939066 PMC7759461

[ref51] Imbalzano G.; Zhuang Y.; Kapil V.; Rossi K.; Engel E. A.; Grasselli F.; Ceriotti M. Uncertainty estimation for molecular dynamics and sampling. J. Chem. Phys. 2021, 154, 074102 10.1063/5.0036522. 33607885

[ref52] Musil F.; Willatt M. J.; Langovoy M. A.; Ceriotti M. Fast and Accurate Uncertainty Estimation in Chemical Machine Learning. J. Chem. Theory Comput. 2019, 15, 906–915. 10.1021/acs.jctc.8b00959. 30605342

[ref53] Alzghoul A.; Alhalaweh A.; Mahlin D.; Bergström C. A. S. Experimental and Computational Prediction of Glass Transition Temperature of Drugs. J. Chem. Inf. Model. 2014, 54, 3396–3403. 10.1021/ci5004834. 25361075

[ref54] DeBoyace K.; Wildfong P. L. The Application of Modeling and Prediction to the Formation and Stability of Amorphous Solid Dispersions. J. Pharm. Sci. 2018, 107, 57–74. 10.1016/j.xphs.2017.03.029. 28389266

[ref55] Ertl P.; Rohde B.; Selzer P. Fast Calculation of Molecular Polar Surface Area as a Sum of Fragment-Based Contributions and Its Application to the Prediction of Drug Transport Properties. J. Med. Chem. 2000, 43, 3714–3717. 10.1021/jm000942e. 11020286

[ref56] Hall L. H.; Kier L. B. The E-State as the Basis for Molecular Structure Space Definition and Structure Similarity. J. Chem. Inf. Comput. Sci. 2000, 40, 784–791. 10.1021/ci990140w. 10850783

[ref57] Pearlman R. S.; Smith K. M. 3D QSAR in Drug Design; Springer: Netherlands, 2002; pp 339–353.

[ref58] Gasteiger J.; Marsili M. Iterative partial equalization of orbital electronegativity—a rapid access to atomic charges. Tetrahedron 1980, 36, 3219–3228. 10.1016/0040-4020(80)80168-2.

[ref59] Gasteiger J.; Marsili M. A new model for calculating atomic charges in molecules. Tetrahedron Lett. 1978, 19, 3181–3184. 10.1016/S0040-4039(01)94977-9.

[ref60] Burden F. R. Molecular identification number for substructure searches. J. Chem. Inf. Comput. Sci. 1989, 29, 225–227. 10.1021/ci00063a011.

[ref61] Nikolic K.; Agababa D. Prediction of hepatic microsomal intrinsic clearance and human clearance values for drugs. J. Mol. Graph. Model. 2009, 28, 245–252. 10.1016/j.jmgm.2009.08.002. 19713138

[ref62] Galvez J.; Garcia-Domenech R.; de Julian-Ortiz J. V.; Soler R. Topological Approach to Drug Design. J. Chem. Inf. Comput. Sci. 1995, 35, 272–284. 10.1021/ci00024a017. 7730417

[ref63] Bartók A. P.; Kondor R.; Csányi G. On representing chemical environments. Phys. Rev. B 2013, 87, 184115 10.1103/physrevb.87.184115.

[ref64] Gillis E. P.; Eastman K. J.; Hill M. D.; Donnelly D. J.; Meanwell N. A. Applications of Fluorine in Medicinal Chemistry. J. Med. Chem. 2015, 58, 8315–8359. 10.1021/acs.jmedchem.5b00258. 26200936

[ref65] Abraham M. H.; Ibrahim A.; Zissimos A. M.; Zhao Y. H.; Comer J.; Reynolds D. P. Application of hydrogen bonding calculations in property based drug design. Drug Discovery Today 2002, 7, 1056–1063. 10.1016/S1359-6446(02)02478-9. 12546895

[ref66] Platts J. A.; Butina D.; Abraham M. H.; Hersey A. Estimation of Molecular Linear Free Energy Relation Descriptors Using a Group Contribution Approach. J. Chem. Inf. Comput. Sci. 1999, 39, 835–845. 10.1021/ci980339t. 10661552

[ref67] Stenzel A.; Goss K.-U.; Endo S. Prediction of partition coefficients for complex environmental contaminants: Validation of COSMOtherm, ABSOLV, and SPARC. Environ. Toxicol. Chem. 2014, 33, 1537–1543. 10.1002/etc.2587. 24668883

[ref68] Brinkmann J.; Exner L.; Luebbert C.; Sadowski G. In-Silico Screening of Lipid-Based Drug Delivery Systems. Pharm. Res. 2020, 37, 249 10.1007/s11095-020-02955-0. 33230602 PMC7683453

[ref69] Brinkmann J.; Huxoll F.; Luebbert C.; Sadowski G. Solubility of pharmaceutical ingredients in triglycerides. Eur. J. Pharm. Biopharm. 2019, 145, 113–120. 10.1016/j.ejpb.2019.10.012. 31682903

[ref70] Pouton C. W. Lipid formulations for oral administration of drugs: non-emulsifying, self-emulsifying and ‘self-microemulsifying’ drug delivery systems. Eur. J. Pharm. Sci. 2000, 11, S93–S98. 10.1016/S0928-0987(00)00167-6. 11033431

