# Supplementary material for: Comparative Analysis of Chemical Descriptors by Machine Learning Reveals Atomistic Insights into Solute–Lipid Interactions
Source: Mol Pharm. 2024 May 23;21(7):3343–55. doi: 10.1021/acs.molpharmaceut.4c00080 (PMC11220795; doi:10.1021/acs.molpharmaceut.4c00080)
Supplement: Supplementary file 1 — mp4c00080_si_001.pdf [file mp4c00080_si_001.pdf]

Supporting Information: Comparative Analysis of Chemical Descriptors by Machine Learning Reveals Atomistic Insights into Solute-Lipid Interactions

Justus Johann Lange,[†] Andrea Anelli,[‡] Jochem Alsenz,[¶] Martin Kuentz,[§] Patrick J. O'Dwyer,[†] Wiebke Saal,[‡] Nicole Wyttenbach,[‡] and Brendan T. Griffin[*,†]

†*School of Pharmacy, University College Cork, College Road, Cork, T12 R229, Cork County, Ireland*

‡*Roche Pharma Research and Early Development, Therapeutic Modalities, Roche Innovation Center Basel, F. Hoffmann-La Roche Ltd., Grenzacherstrasse 124, 4070 Basel, Switzerland*

¶*Independent Researcher, Grenzach-Wyhlen, 79639, Baden-Wuerttemberg, Germany*

§*Insitute of Pharma Technology, University of Applied Sciences and Arts Northwestern Switzerland, Hofackerstrasse 30, Muttenz, CH-4231, Basel City, Switzerland*

E-mail: brendan.griffin@ucc.ie

Phone: +353 (0) 21 490 1657. Fax: +353 (0) 21 490 1656

Supporting Information Available

Model Information Table

Table S1 provides an overview on the derived models and their hyperparameters, which were determined by 10-fold cross-validation on the training set. Non-specified hyperparameters were set to default values.

Table S1: Model information table detailing the parameters of the best model for each descriptor set. Remaining parameters were set to default values.

**Feature**	**Preprocessing**		**Model parameters**			
	**Transformer**	**Scaling**	**Algorithm**	α	**Selection**	**L1 ratio**
2D&3D	n.a.	MinMaxScaler	ElasticNet	0.012	cyclic	0.75
SOAPS	n.a.	StandardScaler	Lasso	0.029	cyclic	n.a.
Abraham	PowerTransformer	MinMaxScaler	Ridge	0.494	n.a.	n.a.
ECFP4	n.a.	n.a.	ElasticNet	0.029	cyclic	0.25

Descriptor Explanation

Table S2 provides an overview on the 15 most influential features for solubility in medium chain triglycerides as determined by the model based on 2D&3D descriptors.

Table S2: Full name and underlying research articles for the 2&3D descriptors calculated by *Mordred.*

Feature	Full name	Reference
TopoPSA	Topological Polar Surface Area	Ertl[S1]
EState_VSA3	Surface contribution to the Electro-Topological State	Hall and Kier[S2]
n5aRing	5-membered aromatic ring count	Moriwaki et al.[S3]
Diameter	Topological Diameter	Moriwaki et al.[S3]
BCUTc-1l	Burden Chemical Abstract Service University of Texas	Pearlman and Smith[S4]
EState_VSA9	Surface contribution to the Electro-Topological State	Hall and Kier[S2]
nHBDon	Number of hydrogen bond donors	Moriwaki et al.[S3]
NsCH3	Number of sCH3	Moriwaki et al.[S3]
TopoPSA(NO)	Topological Polar Surface Area (Accounting for Nitrogen and Oxygen only)	Ertl[S1]
GATS2Z	Geary coefficient of lag 2 weighted by atomic number	Described on p.19 in Todeschini and Consonni[S5]
nBondsD	Number of double bonds in non-kekulized structure	Moriwaki et al.[S3]
PEOE_VSA8	Sum of atomic van der Waals surface area contributions to partial equalization of orbital electronegativities	Gasteiger and Marsili[S6] Labute[S7]
SlogP_VSA1	Sum of atomic van der Waals surface area associated with logP	Labute[S7]
VSA_EState9	Surface contribution to the Electro-Topological State	Hall and Kier[S2]

Uncertainty & Applicability Domain Estimation

Figure S1 provides structural information for the five molecules exhibiting the highest uncertainty per descriptor/model.

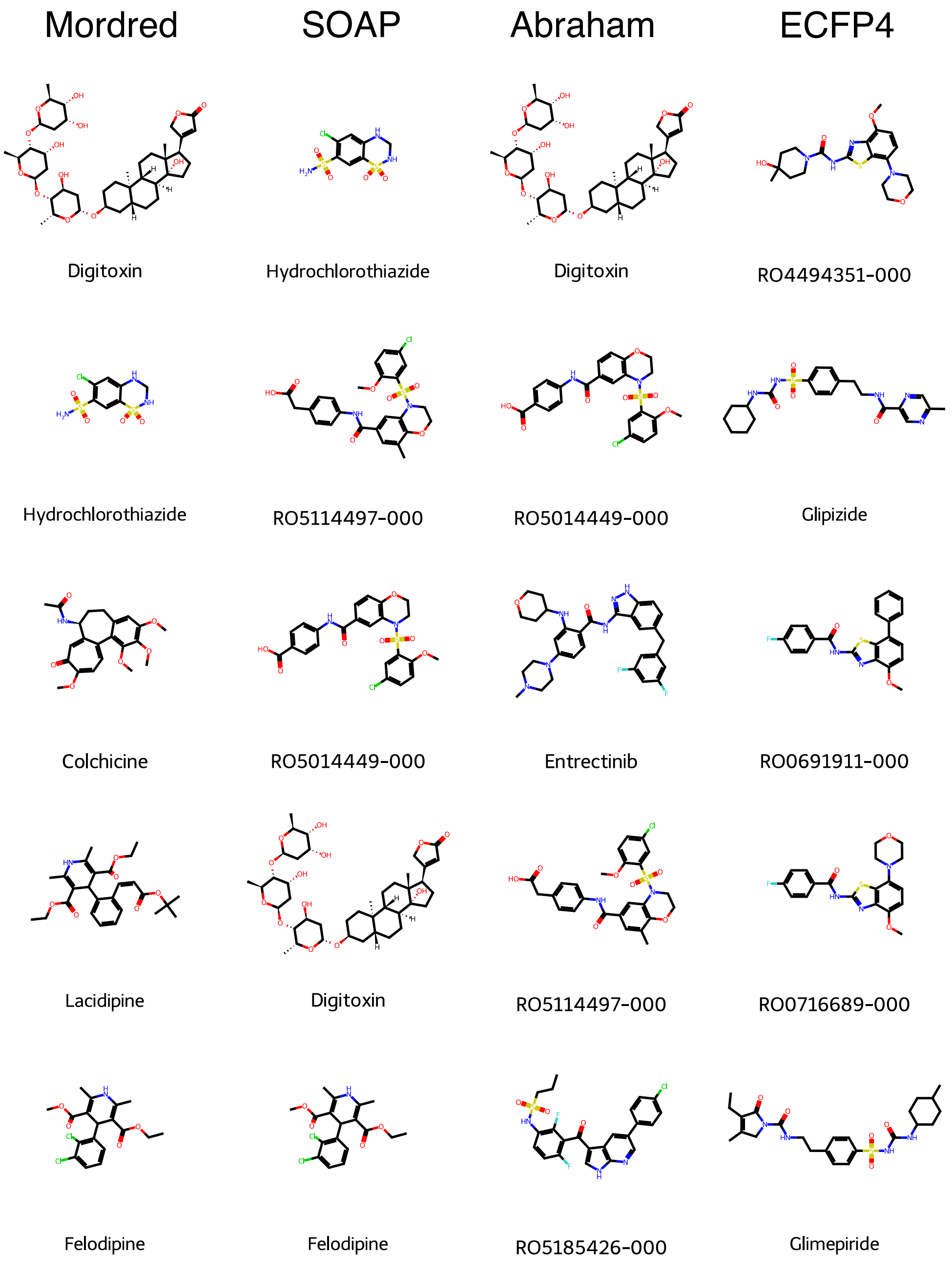

Figure S1: Illustration of the five molecules with the highest uncertainty in the test set for each model/descriptor set.

References

(S1) Ertl, P. *Molecular Drug Properties*; John Wiley & Sons, Ltd, 2007; Chapter 5, pp 111–126.

(S2) Hall, L. H.; Kier, L. B. The E-State as the Basis for Molecular Structure Space Definition and Structure Similarity. *Journal of Chemical Information and Computer Sciences* **2000**, *40*, 784–791.

(S3) Moriwaki, H.; Tian, Y.-S.; Kawashita, N.; Takagi, T. Mordred: a molecular descriptor calculator. *Journal of Cheminformatics* **2018**, *10*.

(S4) Pearlman, R. S.; Smith, K. M. Metric Validation and the Receptor-Relevant Subspace Concept. *Journal of Chemical Information and Computer Sciences* **1999**, *39*, 28–35.

(S5) Todeschini, R.; Consonni, V. *Handbook of Molecular Descriptors*; Wiley, 2000.

(S6) Gasteiger, J.; Marsili, M. Iterative partial equalization of orbital electronegativity—a rapid access to atomic charges. *Tetrahedron* **1980**, *36*, 3219–3228.

(S7) Labute, P. A widely applicable set of descriptors. *Journal of Molecular Graphics and Modelling* **2000**, *18*, 464–477.
